# Difference Analysis of Blood Biochemistry, Slaughter Performance and Gastrointestinal Microbiota in Small-Tailed Han Sheep of Different Sexes

**DOI:** 10.3390/ani16091332

**Published:** 2026-04-27

**Authors:** Mengen Zhang, Rui Han, Anguo Zhang, Chao Xu, Junda Liu, Mengqing Li, Naifeng Zhang, Xunsheng Pang, Shiqin Wang

**Affiliations:** 1 Anhui Province Key Laboratory of Animal Nutritional Regulation and Health, College of Animal Science, Anhui Science and Technology University, Chuzhou 233100, China; 2 Key Laboratory of Feed Biotechnology of the Ministry of Agriculture and Rural Affairs, Feed Research Institute, Chinese Academy of Agricultural Sciences, Beijing 100081, China

**Keywords:** Small-tailed Han sheep, sex, blood biochemical parameters, slaughter performance, gastrointestinal microbiota

## Abstract

This study explored the differences in slaughter performance, blood biochemical indices, and ruminal and colonic microbiota between 6-month-old male and female Small-tailed Han sheep, a typical meat-wool dual-purpose breed in China. Twenty weaned lambs (10 males and 10 females) with uniform body condition were reared under unified feeding management until 6 months of age, followed by slaughter sampling and microbial sequencing detection. Results showed that male lambs had significantly higher pre-slaughter live weight, carcass weight and serum ALP content than females (*p* < 0.05), with lower BUN and β-BHBA levels (*p* < 0.05). High-throughput sequencing of the 16S rDNA gene in rumen fluid and colon contents revealed that microbial alpha diversity in the rumen was extremely significantly higher than that in the colon (*p* < 0.01), and their microbial community structures were distinctly separated (*p* = 0.001). Sex had no significant effect on overall microbial diversity, but altered specific flora and functional pathways: male rumen had higher Actinobacteriota abundance, while female colon had enriched galactose metabolism and male colon had enhanced folate-mediated one-carbon pool pathway. These findings clarify the tissue specificity of gastrointestinal microbiota and sex-related phenotypic differences, providing a theoretical basis for sex-specific feeding of Small-tailed Han sheep.

## Simple Summary

Gastrointestinal microbiota acts as a pivotal hidden organ regulating nutrient metabolism and production performance in ruminants. Small-tailed Han sheep, a renowned meat–wool dual-purpose breed in China, exhibits distinct phenotypic differences driven by sex, yet systematic research on sex-mediated variations in slaughter performance, blood biochemistry, and rumen–colon microbiota at the critical 6-month fattening stage remains limited. This study selected 20 weaned 45-day-old Small-tailed Han sheep (10 males, 10 females) with uniform body condition, reared them under unified management until 6 months of age, and combined slaughter testing, blood biochemical detection, and 16S rDNA high-throughput sequencing to analyze sex differences. Results revealed that male sheep possessed superior slaughter performance and divergent serum biochemical indices compared to females; rumen microbial alpha diversity was significantly higher than that of the colon, with distinct community structure and functional pathways between the two segments. Sex did not affect the overall microbial diversity significantly, but modified the composition of specific microbiota and core metabolic pathways (galactose metabolism in females and folate-mediated one-carbon metabolism in males). These findings confirm the tissue-specific characteristics of gastrointestinal microbiota and sex-mediated phenotypic differences, providing a scientific basis for sex-specific feeding and breeding optimization of Small-tailed Han sheep.

## 1. Introduction

As the core “hidden organ” regulating nutritional metabolism and immunity in ruminants, the gastrointestinal microbiota deeply affects host growth, development, and production performance by participating in nutrient degradation, short-chain fatty acid (SCFA) synthesis, and hormone balance regulation [1,2]. Significant functional differentiation exists along the gastrointestinal tract of ruminants, among which the rumen and colon serve as key fermentation sites with particularly distinct microbiota composition and functions. As the primary fermentation organ, the rumen is enriched with core bacteria such as Bacteroidota and Firmicutes, which efficiently convert roughage into SCFAs (e.g., acetate, propionate) via carbohydrate metabolism and fiber degradation pathways, providing the main energy source for the host [3,4]. The colon undertakes “secondary fermentation” and intestinal barrier maintenance [5,6], with elevated abundances of Ruminococcaceae, Lachnospiraceae (under Firmicutes), Verrucomicrobiota, and other taxa. It specializes in degrading insoluble fiber, producing butyrate, and participating in vitamin synthesis and immune regulation, supporting the host’s secondary utilization of undigested nutrients [3,7]. Such site-specific microbial differences have been confirmed in Small-tailed Han sheep, cattle, and other ruminants, representing a key physiological basis for adapting to high-fiber diets [8,9].

As an excellent dual-purpose meat and wool breed in China, Small-tailed Han sheep exhibits high reproductive performance and rapid growth. Its slaughter performance and blood biochemical parameters directly reflect production potential, while hormone levels indirectly mediate the relationship between microbiota structure and production performance by regulating metabolism and immune function [10,11]. As a core driver of phenotypic differentiation in animals, sex has been shown to affect microbial composition and physiological status in ruminants through sex hormone regulation and divergent energy metabolic demands. For example, male Himalayan tahars have a higher Firmicutes/Bacteroidota ratio, matching their greater energy requirements [8]; male donkeys display significantly higher rumen microbial diversity and elevated abundances of fiber-degrading bacteria compared with females [12]. However, most existing studies on Small-tailed Han sheep have focused on the effects of reproductive stage or feed additives on microbiota and production performance [10,13]. Research systematically investigating the combined effects of sex differences on blood biochemical parameters, slaughter performance, hormone levels, and rumen–colon microbiota in 6-month-old lambs—an essential developmental stage—remains limited.

Current sex-related studies on Small-tailed Han sheep have notable gaps. On the one hand, although functional partitioning between rumen and colon microbiota is well established, the sex-specific differential responses of microbial composition and metabolic pathways (e.g., fiber degradation, SCFA synthesis) in these two gut segments remain unclear. On the other hand, insufficient correlation analysis has been conducted among blood biochemical parameters (e.g., antioxidant enzymes, nutritional metabolic markers), slaughter performance (e.g., average daily gain and carcass traits), hormone levels (e.g., sex hormones and growth hormone), and sex-associated microbiota, making it difficult to reveal the intrinsic regulatory network underlying sex-driven differences in production performance [11,12]. Six-month-old Small-tailed Han sheep are at the early fattening stage, during which physiological and microbial differentiation induced by sex is not yet fully established. Targeted research at this stage can provide critical theoretical support for subsequent sex-specific breeding.

On this basis, the present study took 6-month-old Small-tailed Han sheep as experimental animals. We first clarified the differences in microbial community composition and function between the rumen and colon, and then systematically compared the differentiation characteristics of individuals of different sexes in terms of blood biochemical indices, slaughter performance, hormone levels, and gut microbiota in the two intestinal segments. This study aimed to identify the relationships between sex, physiological phenotypes, and gastrointestinal microbiota, so as to provide a scientific basis for sex-differentiated feeding and management, production performance optimization, and genetic breeding of Small-tailed Han sheep. Meanwhile, it will enrich the basic theoretical system of sex-microbiota-phenotype associations in ruminants.

## 2. Materials and Methods

The Small-tailed Han sheep used in this experiment were provided by the Core Breeding Farm of Small-tailed Han Sheep in Guzhen County, Bengbu City, Anhui Province, China. The animal experiment protocol was approved by the Animal Welfare and Ethics Committee of Anhui Science and Technology University (Approval No.: AK2026075).

A single-factor completely randomized design was used in this study. Twenty 45-day-old weaned Small-tailed Han Sheep with similar weaning weight (10.29 ± 1.96 kg), consistent body condition and unified immunization procedures were selected, consisting of 10 males and 10 females. They were randomly divided into the male group and female group, and raised under unified management until 6 months of age for slaughter and sampling. A 7-day preliminary trial period was set, during which deworming, immunization and environmental acclimatization were completed. During the formal trial period, all sheep were housed in the same standardized fattening shed, where the temperature was controlled at 18~25 °C, relative humidity at 50~70%, with good ventilation and sufficient light, and the stocking density was 1.5 m^2^ per sheep. The experimental diet was formulated as a total mixed ration (TMR) with reference to the Feeding Standard of Meat Sheep (NY/T 816-2021) [14]. The composition and nutritional levels of the diet are shown in Table 1. Sheep had free access to feed and water, and were fed twice a day at fixed times (08:00 and 18:00). Residual feed and manure were cleaned up in a timely manner to keep the pens clean.

### 2.1. Determination Indices and Methods

#### 2.1.1. Blood Biochemical Parameters

Sheep were fasted for 24 h before slaughter. Ten milliliters of jugular vein blood was collected into vacuum blood collection tubes using disposable vacuum blood collection needles, centrifuged at 3500× *g* for 15 min. Serum samples were transferred into 1.5 mL centrifuge tubes and stored at −20 °C for subsequent determination.

Total protein (TP, Catalog Number: HY-50067, Shanghai, China) was determined by biuret colorimetric method, albumin (ALB, Catalog Number: HY-50068, Shanghai, China) by bromocresol green method, alanine aminotransferase (ALT, Catalog Number: HY-50003, Shanghai, China) by ultraviolet-lactate dehydrogenase method, aspartate aminotransferase (AST, Catalog Number: HY-50004, Shanghai, China) by ultraviolet-malate dehydrogenase method, total cholesterol (TC, Catalog Number: HY-50061, Shanghai, China) by oxidase method, triglyceride (TG, Catalog Number: HY-50027, Shanghai, China) by glycerophosphate oxidase method, alkaline phosphatase (ALP, Catalog Number: HY-50005, Shanghai, China) by AMP buffer method, blood urea nitrogen (BUN, Catalog Number: HY-50013, Shanghai, China) by ultraviolet-glutamate dehydrogenase method, glucose (GLU, Catalog Number: HY-50025, Shanghai, China) by oxidase method, β-hydroxybutyric acid (BHBA, Catalog Number: HY-2295, Shanghai, China) content by enzyme-linked immunosorbent assay, and high-density lipoprotein (HDL, Catalog Number: HY-50028, Shanghai, China) and low-density lipoprotein (LDL, Catalog Number: HY-50029, Shanghai, China) by selective surfactant method. The specific operations were performed in accordance with the instructions of Shanghai Kehua Bio-Engineering Co., Ltd., Shanghai China. Serum total antioxidant capacity (T-AOC, Catalog Number: A015-1-2, Shanghai, China), glutathione peroxidase (GSH-Px, Catalog Number: A005-1, Shanghai, China), catalase (CAT, Catalog Number: A007-1-1, Shanghai, China), superoxide dismutase (SOD, Catalog Number: A001-1, Shanghai, China) activities, and malondialdehyde (MDA, Catalog Number: A003-1-2, Shanghai, China) content were determined by colorimetric method, following the instructions of Shanghai Kehua Bio-Engineering Co., Ltd., Shanghai, China.

#### 2.1.2. Slaughter Performance

Pre-slaughter live weight was determined after 24 h of fasting and 2 h of water deprivation. All lambs were slaughtered in the early morning by exsanguination via the carotid artery.

After slaughter, the skin, head, hooves and internal organs of the tested sheep were removed, while the kidneys and perirenal fat were retained. The carcass weight was measured after standing for 30 min. The dressing percentage was calculated as the percentage of carcass weight relative to pre-slaughter live weight. Muscle, adipose tissue and kidneys were stripped from the carcass, and the bone weight was then recorded. The difference between carcass weight and bone weight was defined as net meat weight. The net meat percentage referred to the proportion of net meat weight in pre-slaughter live weight, and the carcass net meat percentage was the ratio of net meat weight to carcass weight. The cross-sectional contour of the eye muscle (Longissimus dorsi) between the 12th and 13th ribs was outlined on sulfate drawing paper to determine the eye muscle area. A vernier caliper was used to measure the tissue thickness at 11 cm from the dorsal midline between the 12th and 13th ribs, which was defined as the GR value. The backfat thickness was measured at the position directly above the midpoint of the eye muscle between the 12th and 13th ribs with a vernier caliper. The tail weight was weighed after tail excision at the anterior edge of the first caudal vertebra.

Meat quality traits: The pH value was determined by inserting the penetration electrode of a portable pH meter (pH-STAR, MAT-THAUS, Eckelsheim, Germany) vertically into the longissimus dorsi muscle of meat samples. Each sample was measured in triplicate, and the average value was calculated. Meat color parameters, including lightness (L*), redness (a*), and yellowness (b*), were detected using a colorimeter (NS-A-1608, Shenzhen Sanenshi Technology Co., Ltd., Shenzhen, China). The cross-section of the longissimus dorsi muscle was selected, with surface fascia and adipose tissue removed. The measuring aperture of the instrument was closely attached vertically to the sample surface. Three distinct detection positions were selected for each sample, and the mean value was recorded. Shear force was measured with a digital muscle tenderness analyzer (C-LM3B, Beijing Tianxiang Feiyu Instrument Equipment Co., Ltd., Beijing, China). Meat cores were collected and sheared along the direction of muscle fibers. Three different sites were determined per sample, and the average value was adopted. Drip loss: Longissimus dorsi muscle was sampled at the thoracolumbar junction, trimmed of fat and connective tissue, and cut into meat strips measuring 5 cm × 3 cm × 2 cm. Each strip was suspended with an S-shaped hook inside a disposable transparent plastic cup without contacting the cup wall, placed into a 20 cm × 14 cm zip-lock bag, sealed tightly, and refrigerated at 4 °C for 24 h. After removal, surface moisture was blotted dry. Drip loss was calculated as the ratio of the weight difference (pre-suspension weight minus post-suspension weight) to the pre-suspension weight. Shear force was determined using a digital muscle tenderness meter (C-LM3B, Beijing Tianxiang Feiyu Instrument Equipment Co., Ltd.). Meat samples were sheared along the direction of muscle fibers, with measurements taken at three distinct sites per sample, and the average value was calculated. Cooked meat percentage: Approximately 100 g of the left psoas major muscle was heated in a water bath at 100 °C for 45 min. After cooling, the cooked meat percentage was calculated based on the weight before and after cooking.

#### 2.1.3. Hormone Indices

Free fatty acid (FFA, Catalog Number: HY-60053, Shanghai, China), insulin (INS, Catalog Number: HY-10069, Shanghai, China), triiodothyronine (T3, Catalog Number: HY-10001, Shanghai, China), thyroxine (T4, Catalog Number: HY-10002, Shanghai, China), cholecystokinin (CCK, Catalog Number: HY-10049, Shanghai, China), growth hormone (GH, Catalog Number: HY-10035, Shanghai, China), estradiol (E2, Catalog Number: HY-10029, Shanghai, China) and testosterone (T, Catalog Number: HY-C0005, Shanghai, China) were determined by double-antibody sandwich enzyme-linked immunosorbent assay, following the instructions of Shanghai Kehua Bio-Engineering Co., Ltd., Shanghai, China.

#### 2.1.4. Rumen and Colon Samples

After slaughter, sheep rumen fluid (from the ventral sac of the rumen) and colonic content samples were collected. The rumen fluid was filtered through 4 layers of sterile gauze to remove feed residues, and the colonic contents were placed in sterile centrifuge tubes. All samples were quickly frozen in liquid nitrogen immediately, transported to the laboratory, and then stored in a −80 °C refrigerator for total microbial DNA extraction.

#### 2.1.5. Total DNA Extraction and 16S rDNA Gene Sequencing Analysis

First, total DNA was extracted from the samples. Genomic DNA of rumen fluid and colonic content samples was extracted using the E.Z.N.A. Stool DNA Kit (Omega Bio-tek, Inc., Norcross, GA, USA) according to the kit instructions. After DNA extraction, the quality and concentration of DNA were detected using a Nanodrop 2000 (Thermo Fisher Scientific, Inc., Wilmington, DE, USA). The V3-V4 region of the bacterial 16S rRNA gene was amplified using universal primers 338F (5′-ACTCCTACGGGAGGCAGCAG-3′) and 806R (5′-GGACTACHVGGGTWTCTAAT-3′). High-throughput sequencing was performed on the Illumina Miseq-PE300 platform, and the detection work was entrusted to Beijing Ovison Gene Technology Co., Ltd., Beijing, China.

#### 2.1.6. Analysis of Microbial Diversity, Composition and Species Differences

Raw sequencing data of fecal microbiota were split into different samples according to Barcode sequences using QIIME1 (v1.8.0) software. Sequencing data were filtered and spliced using Pear (v0.9.6) software. After splicing, high-quality sequences were clustered into operational taxonomic units (OTUs) using the uparse algorithm in Vsearch (v2.7.1) software with a sequence similarity threshold of 97%. OTU representative sequences were aligned with the Silva138 and Unite8.2 databases using the BLAST (v2.15.0) algorithm (e-value threshold = 1 × 10^−5^) to obtain species classification information corresponding to each OTU. Alpha diversity indices were analyzed using QIIME (v1.8.0) software. For species annotation and relative abundance results, species composition histogram analysis was performed using R (v3.6.0) software. For beta diversity, based on OTU and its abundance results, the beta diversity distance matrix was calculated using QIIME (v1.8.0) software, and principal coordinate analysis (PCoA) was plotted using R (v3.6.0) software based on the distance matrix. Linear discriminant analysis effect size (LEfSe) analysis was performed using Python (v2.7) software. Microbial functional prediction: The standardized OTU table was aligned with the KEGG database based on the PICRUSt2010 software(v2.3.0-b) package. According to the alignment information, three levels of metabolic pathway information were obtained. In this experiment, only the differential secondary and tertiary metabolic pathways were analyzed. Microbial functional prediction was only regarded as an exploratory analysis, and the relevant results were used solely for the preliminary exploration of potential biological patterns and correlation trends.

## 3. Statistical Analysis

Data were tested for normality using the Shapiro–Wilk test and for homogeneity of variance using Levene’s test. One-way analysis of variance (ANOVA) was applied for the analysis of slaughter traits, blood biochemical indices, hormone indices, microbial α-diversity, β-diversity and KEGG pathway prediction. Differences in microbial abundance between groups were analyzed using the Kruskal–Wallis test and the Wilcoxon rank-sum test. Correlations between the microbiota and related indicators were analyzed using the Mantel test. *p* < 0.05 was regarded as the threshold for statistical significance. The criteria for the Mantel test were defined as follows: Mantel’s r < 0.2 indicated a weak correlation between two matrices; 0.2 ≤ r ≤ 0.4 indicated a moderate correlation; r > 0.4 indicated a strong correlation. Mantel’s *p* < 0.001 indicated an extremely strong correlation; 0.001 ≤ *p* < 0.01 indicated a significant correlation; 0.01 ≤ *p* < 0.05 indicated a relatively weak correlation; and *p* ≥ 0.05 indicated no significant correlation. The Pearson correlation coefficient (r) ranged from −1 to 1, where r > 0 represented a positive correlation and r < 0 represented a negative correlation.

## 4. Results

### 4.1. Effects of Sex on Slaughter Performance in Small-Tailed Han Sheep

As shown in Table 2, 6-month-old males exhibited significantly higher pre-slaughter live weight, carcass weight, net meat weight, loin eye area, and 1 h post mortem yellowness value compared with females (*p* < 0.05). Net meat percentage and GR value showed an increasing trend in males relative to females (*p* = 0.058, *p* = 0.064). No significant differences were detected for the other measured indices.

### 4.2. Effects of Sex on Blood Biochemical Indices of Small-Tailed Han Sheep

As shown in Table 3, 6-month-old males had higher serum ALP levels than females, whereas serum BUN and β-BHBA concentrations were markedly decreased in males (*p* < 0.05). No differences were found in the other serum parameters.

### 4.3. Effects of Sex on Hormone Indices of Small-Tailed Han Sheep

As shown in Table 4, 6-month-old males had significantly higher serum T levels than females, whereas serum E2 concentrations showed a decreasing trend in males relative to females (*p* = 0.080). No significant differences were detected for the remaining serum parameters.

### 4.4. Effects of Sex on Gastrointestinal Microbiota Diversity in Small-Tailed Han Sheep

In this study, a total of 3,392,857 high-quality sequences were obtained from 40 rumen and colonic content samples of 20 Small-tailed Han sheep using high-throughput sequencing, with an average of 84,821.425 effective sequences per sample. The Goods coverage values of all experimental samples were above 0.97, indicating that the sequencing depth could accurately reflect the microbial composition in the lamb rumen. Clean tags of all samples were clustered (or denoised) at 97% similarity, generating a total of 4973 OTUs. After rarefaction, 4804 OTUs remained, and the representative sequences of OTUs were selected for taxonomic annotation.

**Figure 1:**
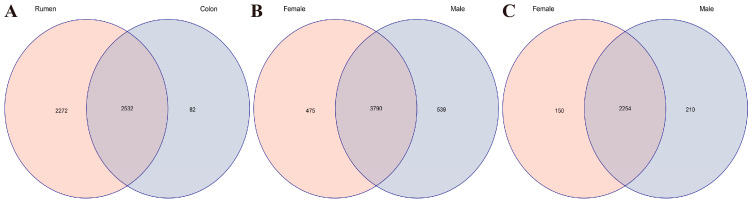
OTU diversity of ruminal and colonic microbiota in 6-month-old Small-tailed Han sheep; (**A**): OTUs in rumen vs. colon; (**B**): OTUs of female vs. male in the rumen; and (**C**): OTUs of female vs. male in the colon.

As shown in Figure 1, a total of 4886 OTUs were obtained from the rumen and colon. Among them, 2272 and 82 OTUs were unique to the rumen and colon, respectively, and 2532 OTUs were shared, accounting for 56.44% of the total OTUs. The shared OTUs accounted for 52.71% of the total OTUs in the rumen, indicating that more than half of the microorganisms in the rumen were also present in the colon. The shared OTUs accounted for 96.86% of the total OTUs in the colon, suggesting that most microorganisms in the colon originated from the rumen. The unique OTUs of the rumen accounted for 47.29% of the total OTUs in the rumen, revealing nearly half of the microorganisms were specific to the rumen and reflecting its functional specificity. In contrast, the unique OTUs of the colon accounted for only 3.14% of the total OTUs in the colon, indicating few unique microorganisms in the colon, whose functions may depend more on the core microbiota.

In total, 4804 ruminal OTUs were detected. Among them, 475 and 539 OTUs were unique to females and males, respectively, and 3790 shared OTUs accounted for 78.89% of the total rumen OTUs. A total of 2614 OTUs were identified in the colon, including 150 unique OTUs in females and 210 unique OTUs in males; the 2254 shared OTUs occupied 86.23% of all colonic OTUs.

As shown in Table 5, the Chao1 index, observed_species, PD_whole_tree and Shannon index of the rumen were significantly higher than those of the colon in 6-month-old Small-tailed Han sheep (*p* < 0.01), indicating significant differences in microbial communities between the rumen and colon.

As shown in Table 6, sex had no significant effect on α-diversity of ruminal and colonic microbiota in Small-tailed Han sheep (*p* > 0.05). Among different tissues, the Chao1 index, observed_species, PD_whole_tree and Shannon index of the rumen were significantly higher than those of the colon (*p* < 0.01), which was consistent with the overall results.

### 4.5. Effects of Sex on β-Diversity of Ruminal and Colonic Microbiota in Small-Tailed Han Sheep

**Figure 2:**
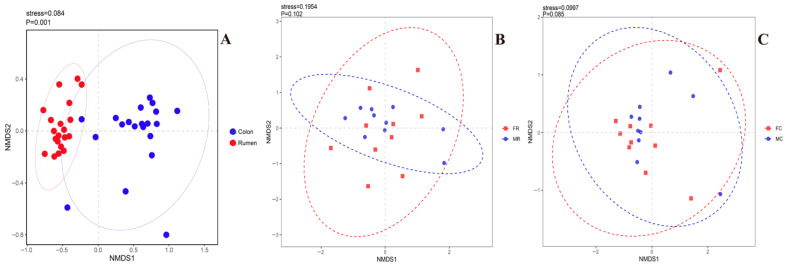
β-diversity of ruminal and colonic microbiota in 6-month-old Small-tailed Han sheep; (**A**): Comparison of β-diversity between rumen and colon; (**B**): β-diversity comparison between females and males in the rumen; and (**C**): β-diversity comparison between females and males in the colon.

Beta diversity analysis was performed based on Bray–Curtis distance to explore differences in community composition among different groups. In the PCoA plot, each point represents a sample, and different colors represent different groups. The closer the distance between samples, the more similar the species composition. As shown in Figure 2A, rumen and colon samples were clearly separated in the NMDS space, indicating a significant difference in microbial community structure between the rumen and colon (*p* = 0.001). Meanwhile, colon samples were more scattered, suggesting higher community heterogeneity than rumen samples.

Figure 2B,C shows the β-diversity distribution of ruminal and colonic microbiota in 6-month-old Small-tailed Han sheep of different sexes. NMDS analysis revealed that ruminal microbial communities of males and females clustered closely with substantial intergroup overlap. No significant difference was found in the β-diversity of ruminal microbiota (*p* = 0.102), indicating a high degree of microbial community consistency between the two sexes. The colonic microbiota showed a marginal difference between females and males (*p* = 0.085).

### 4.6. Effects of Sex on Ruminal and Colonic Microbiota in Small-Tailed Han Sheep

**Figure 3:**
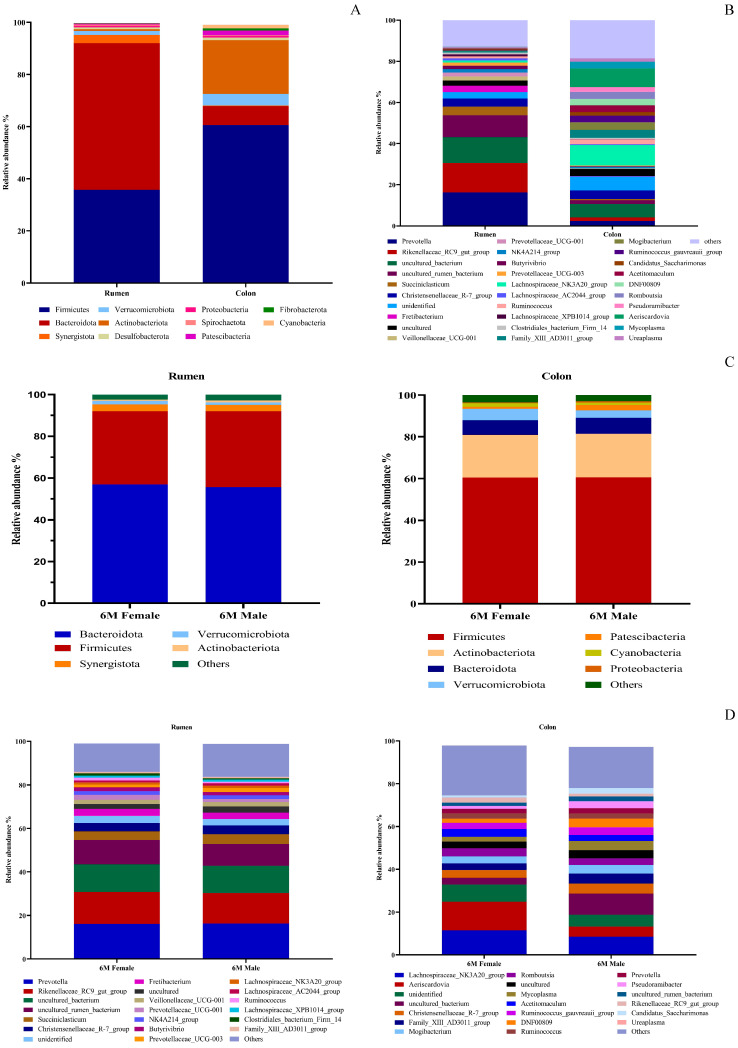
Changes in microbiota at the phylum and genus level in the rumen and colon of 6-month-old Small-tailed Han Sheep. (**A**) Comparison of phylum between rumen and colon; (**B**): Genus-level comparison between females and males in the rumen; (**C**): Phylum-level diversity comparison between females and males in the rumen and colon; (**D**): Genus-level comparison between females and males in the rumen.

As shown in Figure 3A, the dominant phyla in the rumen microbiota of 6-month-old Small-tailed Han sheep were Bacteroidota (56.29%) and Firmicutes (35.75%), whereas the dominant phyla in the colon microbiota were Firmicutes (60.54%) and Actinobacteriota (20.63%).

As shown in Figure 3C, for ruminal microbiota, 6-month-old males had a significantly higher relative abundance of Actinobacteriota than females (*p* < 0.05), while no differences were detected in other phyla (*p* > 0.05). At the phylum level, the colonic microbial composition showed no significant variation between males and females (*p* > 0.05).

As shown in Figure 3B, the relative abundances of the genera Prevotella, Rikenellaceae_RC9_gut_group, Succiniclasticum, Fretibacterium, Veillonellaceae_UCG-001, Prevotellaceae_UCG-001, NK4A214_group, Butyrivibrio, Prevotellaceae_UCG-003, Lachnospiraceae_AC2044_group and Lachnospiraceae_XPB1014_group in the rumen were significantly higher than those in the colon. In contrast, the relative abundances of the genera Lachnospiraceae_NK3A20_group, Ruminococcus, Family_XIII_AD3011_group, Mogibacterium, Ruminococcus_gauvreauii_group, Candidatus_Saccharimonas, Acetitomaculum, DNF00809, Romboutsia, Pseudoramibacter, Aeriscardovia and Ureaplasma in the colon were significantly higher than those in the rumen. There were no significant differences in the relative abundances of Christensenellaceae_R-7_group, Clostridiales_bacterium_Firm_14 and Mycoplasma between the rumen and colon (*p* > 0.05).

As shown in Figure 3D, for the rumen microbiota of 6-month-old lambs, females exhibited higher relative abundances of Ruminococcus and Clostridiales_bacterium_Firm_14 than males, albeit with only trending significance (*p* = 0.060, *p* = 0.065). In contrast, colonic microbiota showed distinct sex-associated differences: Aeriscardovia abundance was significantly elevated in females, DNF00809 was significantly enriched in males, and Pseudoramibacter showed a trending increase in males (*p* = 0.097).

### 4.7. Effects of Sex on KEGG Signaling Pathways in the Rumen and Colon of Small-Tailed Han Sheep

In the rumen, 6 level-1 KEGG metabolic pathways were predicted, namely metabolism, human diseases, genetic information processing, environmental information processing, cellular processes, and organismal systems. a total of 31 level-2 and 163 level-3 KEGG metabolic pathways were predicted in the rumen. In the colon, 6 level-1 KEGG metabolic pathways were also predicted, including metabolism, human diseases, genetic information processing, environmental information processing, cellular processes, and organismal systems. a total of 32 level-2 and 160 level-3 KEGG metabolic pathways were predicted in the colon. Comparative analysis of level-2 pathways between the colon and rumen showed that the colon contained a unique sensory system pathway, which was not detected in the rumen. These differences suggest that the colon is more sensitive to sensing and responding to external stimuli, and its physiological function differs significantly from that of the rumen. The additional sensory system pathway in the colon indicates that it may possess more complex or distinctive mechanisms for perceiving external stimuli. This may be closely related to the colon’s perception and response to the physical properties (e.g., texture and pressure), chemical components (e.g., pH and osmotic pressure) of feed residues, and metabolites of the microbial community during digestion.

**Figure 4:**
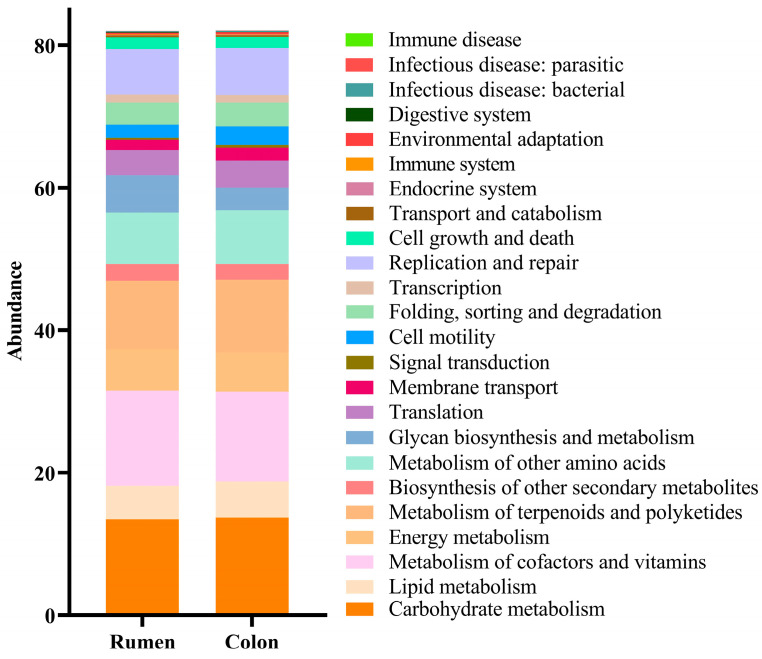
Comparison of level 2 KEGG metabolic pathways in the rumen and colon of 6-month-old Small-tailed Han sheep.

As shown in Figure 4, among level-2 metabolic pathways, the abundances of carbohydrate metabolism, lipid metabolism, metabolism of terpenoids and polyketides, metabolism of other amino acids, translation, membrane transport, signal transduction, cell motility, folding, sorting and degradation, replication and repair, endocrine system, environmental adaptation, infectious disease: bacterial, and immune disease were significantly higher in the colon than in the rumen (*p* < 0.05). In contrast, the abundances of metabolism of cofactors and vitamins, energy metabolism, biosynthesis of other secondary metabolites, glycan biosynthesis and metabolism, transcription, cell growth and death, transport and catabolism, immune system, digestive system, and infectious disease: parasite levels were significantly higher in the rumen than in the colon (*p* < 0.05).

**Figure 5:**
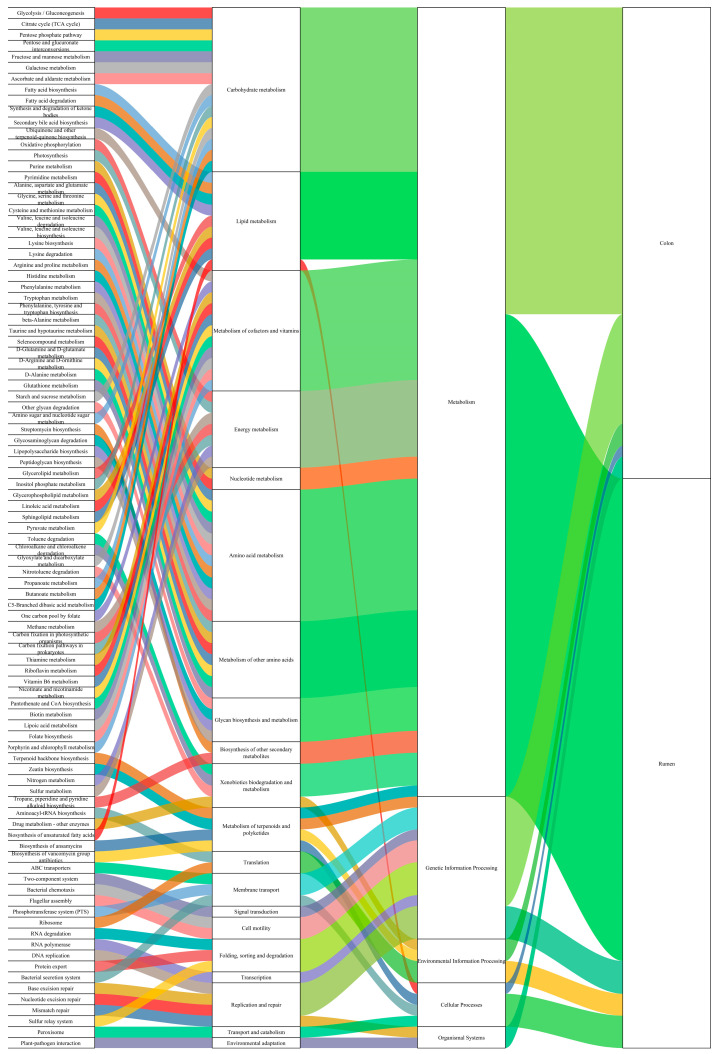
Comparison of KEGG metabolic pathways in the rumen and colon of 6-month-old Small-tailed Han sheep. The width of the connecting lines is proportional to the strength of the correlation coefficient; thicker lines indicate stronger correlations between the connected variables.

As shown in Figure 5, at the level 3 KEGG metabolic pathways, the abundances of citrate cycle (TCA cycle), pentose and glucuronate interconversions, fructose and mannose metabolism, ascorbate and aldarate metabolism, synthesis and degradation of ketone bodies, ubiquinone and other terpenoid quinone biosynthesis, oxidative phosphorylation, glycine, serine and threonine metabolism, valine, leucine and isoleucine degradation, lysine degradation, arginine and proline metabolism, histidine metabolism, phenylalanine metabolism, beta alanine metabolism, taurine and hypotaurine metabolism, selenocompound metabolism, d arginine and d ornithine metabolism, glutathione metabolism, other glycan degradation, amino sugar and nucleotide sugar metabolism, streptomycin biosynthesis, glycosaminoglycan degradation, lipopolysaccharide biosynthesis, sphingolipid metabolism, glyoxylate and dicarboxylate metabolism, one-carbon pool by folate, carbon fixation pathways in prokaryotes, riboflavin metabolism, vitamin B6 metabolism, pantothenate and CoA biosynthesis, lipoic acid metabolism, folate biosynthesis, zeatin biosynthesis, nitrogen metabolism, sulfur metabolism, tropane, piperidine and pyridine alkaloid biosynthesis, drug metabolism—other enzymes, biosynthesis of vancomycin group antibiotics, RNA degradation, RNA polymerase, bacterial secretion system, and peroxisome were significantly higher in the rumen than in the colon (*p* < 0.05). The abundances of glycolysis/gluconeogenesis, pentose phosphate pathway, galactose metabolism, secondary bile acid biosynthesis, purine metabolism, cysteine and methionine metabolism, valine, leucine and isoleucine biosynthesis, lysine biosynthesis, d glutamine and d glutamate metabolism, d alanine metabolism, peptidoglycan biosynthesis, glycerolipid metabolism, glycerophospholipid metabolism, linoleic acid metabolism, pyruvate metabolism, chloroalkane and chloroalkene degradation, nitrotoluene degradation, propanoate metabolism, C5 branched dibasic acid metabolism, methane metabolism, thiamine metabolism, nicotinate and nicotinamide metabolism, terpenoid backbone biosynthesis, aminoacyl tRNA biosynthesis, biosynthesis of ansamycins, ABC transporters, two-component system, bacterial chemotaxis, flagellar assembly, phosphotransferase system (PTS), ribosome, protein export, base excision repair, nucleotide excision repair, sulfur relay system, and plant–pathogen interaction were significantly higher in the colon than in the rumen (*p* < 0.05). Further analysis showed that the level 3 pathways in the rumen were mainly enriched in the level 2 pathways including carbohydrate metabolism, lipid metabolism, metabolism of cofactors and vitamins, energy metabolism, amino acid metabolism, metabolism of other amino acids, glycan biosynthesis and metabolism, biosynthesis of other secondary metabolites, xenobiotic biodegradation and metabolism, metabolism of terpenoids and polyketides, folding, sorting and degradation, transcription, membrane transport, and transport and catabolism. The level 3 pathways in the colon were mainly enriched in the level 2 pathways including carbohydrate metabolism, lipid metabolism, nucleotide metabolism, amino acid metabolism, metabolism of other amino acids, glycan biosynthesis and metabolism, xenobiotic biodegradation and metabolism, energy metabolism, metabolism of cofactors and vitamins, metabolism of terpenoids and polyketides, translation, membrane transport, signal transduction, cell motility, folding, sorting and degradation, replication and repair, and environmental adaptation. Among level 3 pathways, tetracycline biosynthesis, bisphenol degradation, metabolism of xenobiotics by cytochrome P450, biosynthesis of type II polyketide products, spliceosome, phagosome, pathogenic Escherichia coli infection, and toxoplasmosis were unique to the rumen; whereas neomycin, kanamycin and gentamicin biosynthesis, mRNA surveillance pathway, basal transcription factors, plant hormone signal transduction, and olfactory transduction were unique to the colon. These differences reflect the distinct anatomical structures, physiological functions, and innervation between the colon and the rumen.

**Figure 6:**
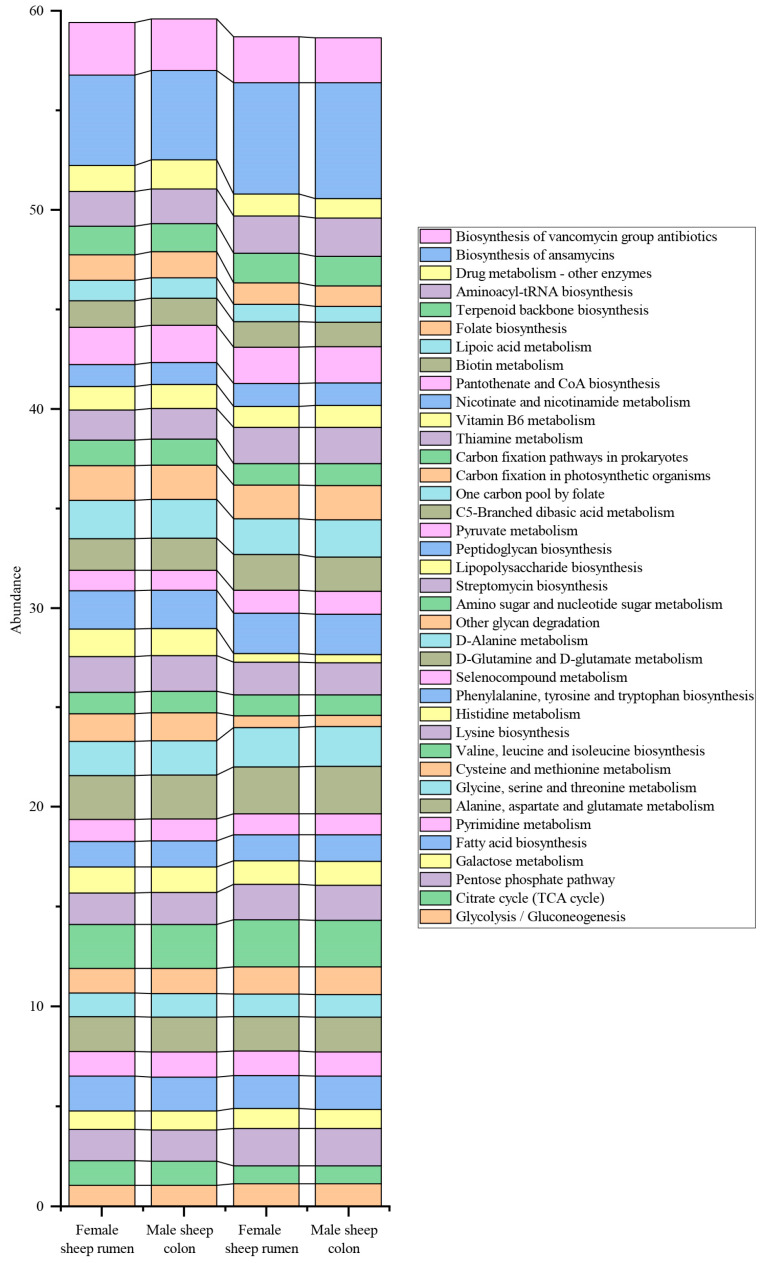
Changes in level-3 metabolic pathways in the rumen and colon of 6-month-old Small-tailed Han sheep.

As shown in Figure 6, at six months of age, the galactose metabolism pathway was significantly enriched in females, while the folate-mediated one-carbon metabolism pathway was markedly enriched in males. No obvious differences in other level-3 metabolic pathways were detected between the two sexes.

### 4.8. Correlation Analysis of Rumen–Colon Microbiota and Correlations Between Microbiota and Slaughter Performance, Blood Biochemical and Hormonal Indices

**Figure 7:**
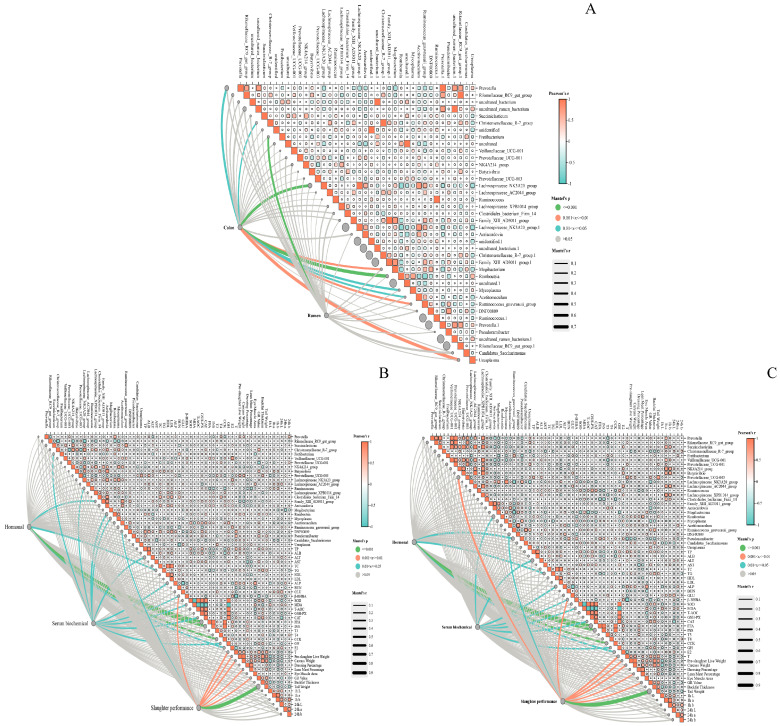
Correlation analysis between rumen and colon microbiota and blood biochemical, hormonal and slaughter performance indices. (**A**) Correlation analysis of bacteria at the genus level in the rumen and colon. (**B**) Correlation analysis between rumen microbiota and blood biochemical, hormonal and slaughter performance indices. (**C**) Correlation analysis between colonic microbiota and blood biochemical, hormonal and slaughter performance indices.

As shown in Figure 7A, Prevotella had a very strong positive correlation with Rikenellaceae_RC9_gut_group, uncultured rumen bacterium, Succiniclasticum, and Christensenellaceae_R-7_group (r > 0.7), suggesting their high co-occurrence in the community and possible sharing of metabolic pathways (e.g., fiber degradation and short-chain fatty acid production) or dependence on the same substrates. Rikenellaceae_RC9_gut_group was also strongly positively correlated with uncultured rumen bacterium and Succiniclasticum (r > 0.6), reflecting their synergistic role in rumen fermentation. Some Lachnospiraceae taxa (such as Lachnospiraceae_AC2044_group and Lachnospiraceae_XPB1014_group) showed moderate negative correlations with Prevotella (r ≈ −0.3~−0.5), indicating possible substrate competition in the rumen (e.g., different preferences for fiber vs. non-fiber carbohydrates). These genera are the functional core of the rumen microecosystem and are highly compatible with rumen fermentation and host metabolism: Prevotella showed the strongest correlation (Mantel’s r ≈ 0.6–0.7), as the most dominant fibrolytic bacterium in the rumen, involved in protein fermentation and short-chain fatty acid production. Rikenellaceae_RC9_gut_group: Correlation r ≈ 0.5–0.6, and was involved in carbohydrate metabolism, synergizing with Prevotella to maintain rumen fermentation homeostasis. Uncultured rumen bacterium: Correlation r ≈ 0.5–0.6, representing key uncultured indigenous microbiota in the rumen that may participate in complex polysaccharide degradation. Succiniclasticum: Correlation r ≈ 0.5, and was mainly involved in succinate metabolism and acts as a key bacterium for butyrate production in rumen fermentation. Christensenellaceae_R-7_group: correlation r ≈ 0.4–0.5, involved in proteolysis and short-chain fatty acid generation, closely related to host nitrogen metabolism in the rumen. Candidatus_Saccharimonas: Correlation r ≈ 0.5, and was involved in rumen glucose metabolism and regulating host energy utilization efficiency. Ureaplasma: Correlation r ≈ 0.4–0.5, and was involved in urea decomposition and maintaining rumen nitrogen balance. The correlation strength of colonic microbiota was weaker than that of rumen microbiota, but the following genera showed tissue specificity: Lachnospiraceae_NK3A20_group: Correlation r ≈ 0.3–0.4, and was involved in colonic fiber degradation and butyrate production, as a core taxon for colonic short-chain fatty acid metabolism. Lachnospiraceae_XPB1014_group: Correlation r ≈ 0.2–0.3, and was related to colonic mucosal barrier function, but with weaker significance and correlation strength than rumen microbiota.

As shown in Figure 7B, slaughter performance was the phenotypic group with the strongest correlation. The following genera showed strong and significant correlations with slaughter indices (pre-slaughter live weight, dressing percentage, lean meat percentage, etc.): Prevotella showed the strongest correlation (Mantel’s r ≈ 0.7–0.8), as the core fibrolytic bacterium in the rumen. Its abundance directly affects host energy acquisition and muscle growth, thereby regulating slaughter performance. Rikenellaceae_RC9_gut_group: Correlation r ≈ 0.6–0.7, and was involved in carbohydrate metabolism and synergizing with Prevotella to maintain rumen fermentation efficiency, indirectly improving slaughter performance. Succiniclasticum: Correlation r ≈ 0.5–0.6, and affects host energy utilization efficiency by regulating succinate metabolism and butyrate production, thereby influencing slaughter indices such as lean meat percentage. Christensenellaceae_R-7_group: Correlation r ≈ 0.5–0.6, and was involved in proteolysis and short-chain fatty acid production, closely related to host nitrogen metabolism and muscle synthesis. Candidatus_Saccharimonas: Correlation r ≈ 0.5, and was involved in rumen glucose metabolism and regulating host energy utilization efficiency, and was positively correlated with dressing percentage. Serum biochemical indices showed a moderate correlation strength with the microbiota. The following genera exhibited significant correlations: Lachnospiraceae_NK3A20_group: Correlation r ≈ 0.4–0.5, and was involved in colonic fiber degradation and butyrate production. Its abundance was significantly correlated with serum lipid and protein metabolic indices (e.g., ALB, TP, and TG). Lachnospiraceae_XPB1014_group: Correlation r ≈ 0.3–0.4, and was weakly correlated with serum inflammatory indices (e.g., IL-1β and TNF-α) and lipid metabolic indices (e.g., HDL and LDL), reflecting the regulatory role of the microbiota in host metabolic homeostasis. Correlations with hormonal indices (gray/cyan lines, *p* > 0.05 or r < 0.3). Hormonal indices showed the weakest correlation with the microbiota. Only a few genera presented weak correlations (r < 0.3, *p* > 0.05). For example, some Lachnospiraceae taxa had very weak positive correlations with growth hormone (GH) and insulin (INS), suggesting that the direct regulatory effect of the microbiota on host hormones is limited, and the influence is mostly indirect through metabolism.

As shown in Figure 7C, strong correlations with slaughter performance (orange/green thick lines, *p* < 0.001 or 0.001 < *p* ≤ 0.01). Slaughter performance was the phenotypic group with the strongest correlation. The following genera showed extremely strong correlations with pre-slaughter live weight, carcass weight, dressing percentage, lean meat percentage and other indices: Prevotella showed the strongest correlation (Mantel’s r ≈ 0.8–0.9, thickest line). As the core fibrolytic bacterium in the rumen, its abundance directly affects host energy acquisition and muscle growth, making it a key taxon regulating slaughter performance. Rikenellaceae_RC9_gut_group: Correlation r ≈ 0.7–0.8, and was involved in carbohydrate metabolism and synergizing with Prevotella to maintain rumen fermentation efficiency, thereby indirectly improving slaughter performance. Succiniclasticum: Correlation r ≈ 0.6–0.7, and affects host energy utilization efficiency by regulating succinate metabolism and butyrate production, thus influencing slaughter indices such as lean meat percentage. Christensenellaceae_R-7_group: Correlation r ≈ 0.6–0.7, and was involved in proteolysis and short-chain fatty acid production, closely related to host nitrogen metabolism and muscle synthesis. Prevotellaceae_UCG-001/003, NK4A214_group: Correlation r ≈ 0.5–0.6, and acts synergistically with Prevotella to promote rumen fermentation and host growth. Candidatus_Saccharimonas, Ureaplasma: Correlation r ≈ 0.5–0.6, and was involved in the regulation of rumen glucose metabolism and nitrogen balance, indirectly affecting slaughter performance. Serum biochemical indices showed a moderate correlation strength with the microbiota. The following genera were significantly correlated with indices related to metabolic homeostasis: Lachnospiraceae_NK3A20_group: Correlation r ≈ 0.4–0.5, and was involved in colonic fiber degradation and butyrate production. Its abundance was significantly correlated with serum protein metabolism (ALB, TP) and lipid metabolism (TG, HDL) indices. Lachnospiraceae_XPB1014_group: Correlation r ≈ 0.3–0.4, and was weakly correlated with serum inflammatory indices (e.g., SOD, MDA) and lipid metabolism indices (e.g., LDL), reflecting the regulatory role of the microbiota in host metabolic homeostasis. Clostridiales_bacterium_Firm_14: Correlation r ≈ 0.3–0.4, and was associated with serum nitrogen metabolism and energy metabolism indices such as blood urea nitrogen (BUN) and glucose (GLU). Correlations between hormonal indices and the microbiota were relatively weak, and only a few genera showed significant weak correlations: Prevotella: Correlation r ≈ 0.3–0.4 (orange line, *p* < 0.01), and was weakly correlated with growth hormone (GH), insulin (INS), estradiol (E2) and other hormones, suggesting that the microbiota indirectly affects hormone levels through metabolism, with limited direct regulatory effects. Rikenellaceae_RC9_gut_group: Correlation r ≈ 0.2–0.3 (gray line, *p* > 0.05), with no significant correlation with hormonal indices, indicating a weak direct regulatory effect on hormones.

## 5. Discussion

### 5.1. Effects of Sex on Slaughter Performance of Small-Tailed Han Sheep

In ruminant production, sex, as an important physiological regulatory factor, has been confirmed to affect slaughter performance and meat quality in studies of various breeds and ages. However, the patterns of its effects show certain specificity due to differences in breed genetic characteristics, growth stages and feeding conditions. In terms of slaughter performance, males of most breeds exhibit advantages in growth-related traits, and 12-month-old male Nanchang Black Goats had extremely significantly higher carcass weight, net meat weight, bone weight and loin eye area than females, while production efficiency indicators such as dressing percentage and net meat percentage were also significantly superior to those of females [15]; the pre-slaughter live weight, carcass weight and net meat weight of 7-month-old Dorset × Small-tailed Han sheep F1 male lambs were significantly higher than those of female lambs [16]; In 12-month-old Bamei Mutton sheep, males exhibited superior performance to females in carcass weight, carcass size, and loin eye area [17]; 8-month-old Hu sheep male also showed a significant sexual advantage in loin eye area [18]. However, exceptions exist: 6-month-old Sunite sheep females had significantly greater net meat weight and net meat percentage, whereas males only showed advantages in bone weight and bone-to-meat ratio [19], reflecting breed-specific sexual dimorphism. In terms of meat quality indices, the differential characteristics are more diverse, as the shear force related to tenderness was extremely significantly higher in 12-month-old male Nanchang Black Goats than in females, and the shear force and cooking loss rate of 12-month-old and growing Bamei Mutton sheep as well as 8-month-old male Hu sheep were also significantly higher than those of females, indicating that females have an advantage in meat tenderness [18,19,20]. Cooked meat percentage showed breed differentiation, being significantly higher in 12-month-old female Nanchang Black Goats than in males [15], while significantly lower in female growing Bamei Mutton sheep than in males [20]; sexual differences in meat color and pH were relatively inconsistent: the L* value of 12-month-old male Bamei Mutton sheep was significantly higher than that of females [18], and the L* value, b* value and pH at 24 h post mortem of Gentile di Puglia male lambs were significantly higher than those of female lambs [21], but no significant sexual differences in pH and meat color indices were observed in multiple breeds such as Nanchang Black Goat, Sunite sheep and Hu sheep [15,16,19]. The effect of sex on drip loss also varied by breed, being significantly lower in female Sunite sheep than in males [16], while no significant differences were found in some breeds [15,17]. In the present study, 6-month-old Small-tailed Han sheep were used to further verify the effect of sex on the production performance of ruminants. The results showed that the pre-slaughter live weight, carcass weight, loin eye area and yellowness value at 1 h post mortem of males were significantly higher than those of females, and the net meat percentage and GR value also tended to be higher in males than in females. This is consistent with the advantageous characteristics of males in growth performance and some meat color indices in most breeds, and also provides data support for supplementing the sexual difference patterns of ruminants of different breeds and ages.

### 5.2. Effects of Sex on Blood Biochemical and Hormonal Indices of Small-Tailed Han Sheep

Blood serves as the core carrier for material transport and metabolic regulation in the body. Serum biochemical indicators can directly reflect the nutritional metabolic status, organ function and overall health of ruminants. The differentiation of hormone profiles caused by sex (e.g., the dominant roles of testosterone and estrogen) usually leads to breed-specific patterns in serum biochemical indicators by regulating metabolic pathways. In terms of serum protein and nitrogen metabolism-related indicators, sex differences in various species have been widely confirmed: in 1-year-old native Nepalese sheep, TP was significantly higher in males, while ALB was significantly higher in females [22]; BUN concentration was significantly higher in males, and LDL content was higher in females [23]; glucose, total cholesterol and urea nitrogen were significantly higher in male horses than in females [24], and this trend also exists in poultry such as broilers and chicks—serum total cholesterol, TG and urea contents are generally higher in roosters than in hens [25]. However, there are exceptions: in green-legged partridge chicks, total cholesterol and triglyceride were significantly higher in females, and urea content was also numerically higher [26], suggesting that sex differences in nitrogen and lipid metabolism-related indicators may be regulated by species genetic characteristics. In terms of enzyme activities and energy metabolism intermediates, ALP is a key indicator reflecting metabolic intensity, growth performance and skeletal development, and its activity is closely related to the absorption and utilization of carbohydrates, fats and proteins [27]. It has been confirmed that castration can up-regulate ALP activity and promote fat synthesis [28]. In the present study, serum ALP content in 6-month-old male Small-tailed Han sheep was significantly higher than that in females, and all values were within the normal reference range (93–387 U/L) [29]. This result not only indicates that males have a faster growth rate and higher metabolic intensity, but also implies that their osteocyte maturation and skeletal calcification progress earlier than females, which is consistent with the superior growth performance of males in pre-slaughter live weight, carcass weight and loin eye area mentioned above. As a key intermediate of fatty acid metabolism in the liver, serum β-BHBA content can directly reflect fat mobilization and energy metabolism status. In this study, serum β-BHBA content was significantly higher in females than in males, which was presumed to be related to the increased secretion of estrogen and progesterone after females reach sexual maturity; estrogen can reduce insulin sensitivity, promote fat mobilization, and enhance hepatic ketogenesis, thereby leading to β-BHBA accumulation [30]. This may be an adaptive strategy for females to reserve energy for subsequent reproductive cycles, such as estrus and pregnancy. In contrast, males of the same age are dominated by testosterone secretion, which can reduce β-BHBA production by enhancing insulin sensitivity and inhibiting lipolysis [31]. Essentially, the sex differences in serum biochemical indicators of sheep are derived from sex-determined sex hormone profile differentiation: males are dominated by testosterone, and females by estrogen and progesterone, with extremely low levels of the opposite sex hormones [32]. These two types of hormones eventually lead to species-specific sex patterns in key indicators such as ALP, β-BHBA and BUN by regulating insulin sensitivity, fat metabolism pathways and protein synthesis efficiency. The characteristics of significantly increased ALP and significantly decreased β-BHBA and BUN in 6-month-old Small-tailed Han sheep males in this study are consistent with the common trend of higher metabolic intensity in males of most species, and also provide data support for revealing the sex-specific metabolic mechanism of sheep and optimizing precise feeding schemes for lambs of different sexes.

### 5.3. Effects of Sex on Gastrointestinal Microbiota Richness and Diversity of Small-Tailed Han Sheep

In recent years, the association between sex and slaughter performance, and meat quality in ruminants has been fully confirmed, but the regulatory mechanism of sex differences in microbial communities in core gastrointestinal segments (rumen and colon) still lacks systematic analysis. This research gap limits the in-depth understanding of sex-specific metabolism and production performance in ruminants. Rumen and intestinal microorganisms are key symbiotic partners of the host: the host provides a stable living environment and nutritional substrates for microorganisms, while microorganisms produce volatile fatty acids, vitamins and other metabolites through feed fermentation, which provides the main energy source for ruminants and deeply participates in the digestion, metabolism and absorption of carbohydrates, proteins and lipids. Most current studies focus on the regulation of external factors, such as diet and rearing environment, on gastrointestinal microorganisms, but often ignore the potential role of sex as an intrinsic physiological trait. Compared with the extensive research on sex and intestinal microbiota in humans and rodents [33,34], relevant explorations in ruminants are still scarce; existing studies mostly focus on fecal microorganisms, while research on sex differences in the two core fermentation sites (rumen and colon) is particularly rare.

As core indicators reflecting the richness and evenness of microbial communities, Chao1 and Observed species in α-diversity mainly characterize species richness, while PD whole tree and Shannon index are related to community evenness. As the core site of feed fermentation in ruminants, rumen microorganisms (e.g., Bacteroides and Ruminococcus) can efficiently decompose complex carbohydrates [35]; while the fermentation substrates of the colon are mostly crude fiber, resistant starch and unabsorbed small molecular sugars remain undegraded by the rumen, with significantly lower digestion efficiency than the rumen [36]. Current studies on the comparison of microbial diversity between rumen and colon are still controversial: some studies found that α-diversity of fecal microorganisms (mainly derived from colon) was higher than that of rumen in Tibetan sheep and Boer goats [37,38]. However, the results of this study showed that Chao1 index, Observed species, PD whole tree and Shannon index in the rumen of Small-tailed Han sheep were significantly higher than those in the colon, which is consistent with the findings in suckling Angus heifers [39] and steers [9], suggesting that the unique fermentation environment of the rumen (e.g., substrate availability and pH stability) is more conducive to the enrichment and evenness of microbial communities.

As a key intrinsic factor affecting intestinal microbial composition, the regulatory pattern of sex on ruminant microbiota has not reached a consensus. Studies have shown that there is no significant difference in α-diversity of microbiota between male and female Qianbei Ma sheep and Weining sheep, but the Shannon index of male black goats is higher than that of females, and the β-diversity of male and female microbiota is clearly separated in Qianbei Ma sheep and black goats [40]. The Shannon index of male river buffaloes during the growing period is significantly higher than that of females [41]; although there is no difference in α-diversity between male and female Hanwoo cattle at the early fattening stage, significant differentiation exists in microbial community structure and functional gene profiles [12]. In the present study, sex had no significant statistical effect on the α-diversity of rumen and colonic microbiota in 6-month-old Small-tailed Han sheep, but all α-diversity indices of males were numerically higher than those of females, implying that the gastrointestinal microbiota of males may have higher species richness or community complexity This difference is presumed to be related to sex-specific physiological characteristics (e.g., metabolic intensity and intestinal structure) and hormone levels (e.g., regulation of metabolic pathways by testosterone)—testosterone may indirectly shape microbial community structure by affecting intestinal mucosal environment or nutrient absorption efficiency, but the specific driving mechanism still needs to be further verified by combined analysis of microbial functional metabolism and host hormone profile.

### 5.4. Effects of Sex on Gastrointestinal Microbiota Composition of Small-Tailed Han Sheep

In the present study, 16S rDNA gene MiSeq sequencing was used to systematically analyze the microbiota structure in the rumen and colon of 6-month-old Small-tailed Han sheep and the regulatory role of sex on gastrointestinal microbial communities. The colonization and development of mammalian gastrointestinal microbiota show obvious temporal dynamics, and unique dominant microbiota adapted to functional requirements are formed at different physiological stages. At the phylum level, Firmicutes, Bacteroidota and Verrucomicrobiota were the core dominant phyla in the rumen and colon of Small-tailed Han sheep, which was consistent with the findings in ruminants such as Tibetan sheep [37], Hu sheep [42] and beef calves [39], reflecting the conservation of gastrointestinal microbiota composition in ruminants. The present study found that the abundances of Bacteroidota, Synergistota, Patescibacteria and Cyanobacteria in the rumen of Small-tailed Han sheep were significantly higher than those in the colon, while Firmicutes, Verrucomicrobiota and Actinobacteriota were significantly enriched in the colon. This trend was similar to that in 8-month-old Liaoning Cashmere goats [43], confirming the inherent differences in the abundance of dominant microbiota in different gastrointestinal segments of ruminants. The core driving factors are segment-specific physiological functions, substrate types and microenvironmental characteristics. From the perspective of functional adaptation, the rumen, as a unique “high-efficiency fermenter” of ruminants, has the core function of degrading complex carbohydrates and proteins in feed. Its neutral to slightly alkaline anaerobic microenvironment provides suitable conditions for functional microbiota: Bacteroidota is rich in glycoside hydrolase and polysaccharide lyase genes in its genome, and is the core microbiota for the hydrolysis of complex polysaccharides and proteins [4]; Synergistota can convert free amino acids into intermediates by encoding amino acid reductive degrading enzymes, providing substrates for syntrophic bacteria and methanogens [44]; Patescibacteria can assist in maintaining the stability of microbiota structure, participate in mineral transport, and are positively correlated with ruminal volatile fatty acid (VFA) concentration [13]. The high abundance of these phyla is highly compatible with the abundant fermentable substrates and microenvironment in the rumen. The core function of the colon is to complete the “secondary fermentation” of residual substrates, absorb water and electrolytes, and maintain intestinal barrier homeostasis. Its acidic, low redox potential and high mucus layer microenvironment are more suitable for specific microbiota [5,6]: Firmicutes can secrete cellulase to degrade insoluble fiber and produce butyrate, supplying energy for colonic epithelial cells [3]; Verrucomicrobiota excels in degrading mucins and promoting the expression of intestinal tight junction proteins [7]; Actinobacteriota can synthesize essential nutrients, inhibit potential pathogens, and participate in residual polysaccharide degradation [45,46]. These functions precisely match the physiological requirements of the colon. As an intrinsic physiological regulatory factor, sex shows significant diversity in its effects on gastrointestinal microbiota at the phylum level in different ruminant species. For example, the abundances of Fibrobacterota and Desulfobacterota were significantly higher in male black goats than in females [40], Verrucomicrobiota was more enriched in male river buffaloes during the growing period [41], and Firmicutes in feces were more abundant in bulls when the diet changed [47]. However, in the present study, only the abundance of Actinobacteriota in the rumen of 6-month-old males was significantly higher than that of females, with no significant sex differences in other phyla. This was inconsistent with the findings that Actinobacteriota were more enriched in females in Weining sheep [40] and Dezhou donkeys [12]. Such differences are presumed to be related to breed genetic characteristics, physiological stage (6 months of age, not fully sexually mature), and consistent feeding environment: as a meat-type breed, the metabolic intensity and growth performance of 6-month-old Small-tailed Han sheep males were significantly higher than those of females. Testosterone may indirectly affect the colonization of Actinobacteriota by regulating the ruminal mucosal environment or nutrient absorption efficiency. The inherent differences in hormone regulation patterns and intestinal physiological structure among breeds may lead to breed-specific effects of sex on microbiota. However, the specific regulatory mechanism (e.g., the interaction pathway of hormone–intestinal environment–microbiota) still needs to be further verified by multi-omics techniques such as metabolomics and transcriptomics.

At the genus level, the present study found that Prevotella, Rikenellaceae_RC9_gut_group, Lachnospiraceae_NK3A20_group, Aeriscardovia and Christensenellaceae_R-7_group were the dominant intestinal genera, which were consistent with the findings of Wang et al. [48], indicating that these genera play core roles in the intestinal microecosystem of ruminants and their dominant status is universal. Due to the differences in physiological functions, the composition and functional emphasis of dominant genera vary significantly between different segments. Rumen microbiota mainly focuses on the initial degradation and metabolism of dietary nutrients, while colonic microbiota pays more attention to the maintenance of intestinal health, subsequent nutrient transformation and immune regulation. The abundances of Prevotella, Prevotellaceae_UCG-001 and Prevotellaceae_UCG-003 (Bacteroidaceae) in the rumen were significantly higher than those in the colon. The three genera synergistically participate in carbohydrate degradation and lipid metabolism, maintain ruminal lipid metabolism homeostasis, and improve the host’s dietary adaptability [49]. Among them, Prevotella degrades non-fiber carbohydrates and proteins, indirectly supporting lipid metabolism; Prevotellaceae_UCG-001 participates in cellulose degradation and odd/branched-chain fatty acid synthesis, regulating microbial membrane fluidity; Prevotellaceae_UCG-003 participates in polyunsaturated fatty acid hydrogenation and complex polysaccharide degradation, providing precursors for the synthesis of specific fatty acids [49]. The abundances of Lachnospiraceae_AC2044_group and Lachnospiraceae_XPB1014_group (Lachnospiraceae and Firmicutes) in the rumen were significantly higher than those in the colon. Both are involved in carbohydrate metabolism but have different functional emphases. Lachnospiraceae_AC2044_group contains [FeFe]-hydrogenase and produces hydrogen during fermentation. Its abundance is significantly negatively correlated with exhaled methane levels, providing a theoretical reference for methane mitigation in ruminants [50]. Lachnospiraceae_XPB1014_group is a SCFA-producing bacterium that focuses on carbohydrate fermentation and cellulose degradation, decomposes complex polysaccharides, and has anti-inflammatory activity to maintain ruminal homeostasis [51]. The abundances of another five genera, such as Succiniclasticum, in the rumen were also significantly higher than those in the colon. Their functions and abundance advantages are as follows: Succiniclasticum converts succinate to propionate (a precursor for gluconeogenesis), and its increased abundance is closely related to the enrichment of Prevotella, which can provide sufficient succinate [52]; Fretibacterium is an obligate anaerobe, and combined with the function of its phylum Synergistota, it is presumed to play a synergistic metabolic role in the rumen [44]; Veillonellaceae_UCG-001 belongs to Firmicutes and is presumed to be a ruminal fibrolytic bacterium participating in the initial degradation of dietary fiber [53]; Butyrivibrio is a core butyrate-synthesizing genus, and its high abundance indicates a wide range of ruminal fermentation substrates, facilitating host nutrient absorption and transformation [54]; and NK4A214_group can degrade refractory polysaccharides and fiber, and concentrate supplement can increase its proportion. Its abundance is dominant in the rumen due to direct contact with concentrate [55]. Different from rumen microbiota, the abundances of Lachnospiraceae_NK3A20_group, Ruminococcus, Family_XIII_AD3011_group, Mogibacterium, Ruminococcus_gauvreauii_group, Candidatus_Saccharimonas, Acetitomaculum, DNF00809, Romboutsia, Pseudoramibacter, Aeriscardovia, and Ureaplasma in the colon were significantly higher than those in the rumen. These genera are mainly involved in maintaining intestinal health, subsequent fiber degradation, immune metabolism and inflammatory regulation. Lachnospiraceae_NK3A20_group belongs to Lachnospiraceae (Firmicutes). Different from the functionally dominant genera of the same family in the rumen, it relies on oligosaccharides produced by other microorganisms as carbon sources, and ferments to produce SCFAs and ATP for the host. Its enrichment in the colon is related to the high accumulation of oligosaccharides in the colon [56]. Christensenellaceae_R-7_group belongs to Firmicutes, which can degrade cellulose and hemicellulose into SCFAs, maintain intestinal microecological balance, and is associated with intestinal health [57]. Ruminococcus and Ruminococcus_gauvreauii_group both belong to Ruminococcaceae and are significantly enriched in the colon. The core reason is that the colon, as the main metabolic site of carbohydrates, can provide sufficient fermentation substrates. Among them, Ruminococcus contains abundant degrading enzymes and can enhance fiber degradation ability [58]; Ruminococcus_gauvreauii_group has multiple characteristics and can synergize with R. bili to adapt to the biliary environment, and its enrichment may be related to colonic bile acid metabolism [58]. The enrichment of Family_XIII_AD3011_group in the colon is consistent with the findings in goats during gestation and lactation. This genus is closely related to fiber-degrading taxa and may participate in hormone homeostasis regulation by reducing intestinal E2/Prog concentrations (negative correlation), alleviating intestinal barrier damage and maintaining colonic health [59]. Mogibacterium is an obligate anaerobe that metabolizes simple carbohydrates and amino acids. Its abundance in the colon is significantly higher than that in the rumen, which is consistent with previous studies [60]. However, this genus has dual effects: it can inhibit anti-tumor immunity and is associated with colorectal cancer. Its specific effects on colonic health in ruminants still need to be explored [60]. The enrichment of four genera including Candidatus_Saccharimonas in the colon is related to their own functions and colonic physiological environment: Candidatus_Saccharimonas is significantly increased in dairy goats fed a low-fiber diet and is beneficial to immune recovery in immunodeficient patients [61,62], so it is presumed to participate in host dietary fiber metabolism and intestinal immune regulation; Acetitomaculum produces acetate, participates in carbohydrate fermentation, supplies energy and maintains intestinal homeostasis, and its enrichment in the colon is similar to previous studies [63]; DNF00809 is closely related to the occurrence of subacute ruminal acidosis (SARA), and its enrichment may aggravate intestinal disorders and promote inflammation, echoing the conclusion that “the intestine is the main anti-inflammatory site” [64]; and Pseudoramibacter has fiber degradation potential, and its enrichment in the colon indicates that it may participate in anaerobic fiber decomposition in the colon [65]. Aeriscardovia belongs to Bifidobacteriaceae (Actinobacteriota), which can synthesize a variety of enzymes, amino acids and vitamins, and metabolize acetate and lactate [66]. When it was enriched in the colon, the abundances of Lachnospiraceae_NK3A20_group and Candidatus_Saccharimonas also increased significantly, which was consistent with the conclusion that the three genera are significantly positively correlated [62].

Intestinal microbiota is significantly affected by host sex, and sex-biased bacteria regulate metabolism. Among them, Ruminococcus (an important fibrolytic bacterium in the rumen) is closely associated with sex regulation. Its core function is to participate in cellulose degradation and acetate production, playing a key role in carbohydrate metabolism. Previous studies have confirmed that it is a typical sex-biased bacterium in the pig intestine, related to skatole production and fat deposition, and its abundance is regulated by sex hormones. The abundance of Ruminococcus was significantly higher in intact boars than in sows, and shifted to the sow microbiota characteristics after castration, confirming that it is an androgen-dependent bacterium [67]. Mouse experiments also confirmed that its abundance was significantly higher in males than in females [68]. However, studies on wild blue sheep in Helan Mountain showed that there was no difference in the abundance of Ruminococcaceae between male and female blue sheep in summer, and both were higher than those in the winter group [69], suggesting that its abundance is regulated by multiple factors. In the present study, the abundance of Ruminococcus in the rumen of females tended to be higher than that of males, which was consistent with the findings of Guo et al. [35]. It is presumed that the increased abundance can enhance fiber fermentation efficiency, produce more SCFAs, promote the high expression of transport genes and accelerate energy supply. This echoes the previous result that serum glucose in females was numerically higher than that in males, indicating that it may affect host physiology by regulating nutrient metabolism. Clostridiales_bacterium_Firm_14 belongs to Clostridiales (Firmicutes). Studies on grazing yaks during the cold season showed that it is related to energy metabolism, cellulose degradation, and adaptation to cold stress, and its ruminal abundance increased significantly in the cold season [70]. In the present study, the abundance of this genus in the rumen of females tended to be higher than that of males. Since the feeding conditions of male and female sheep were consistent, excluding environmental and dietary interference, the reasons and specific functions of their sex-related abundance difference still need in-depth research. In addition, the abundance of Aeriscardovia in the colon of females was significantly higher than that of males. Combined with the above, this genus is significantly positively correlated with Lachnospiraceae_NK3A20_group and Candidatus_Saccharimonas in the colon [62]. In the present study, the abundances of the latter two genera in the colon of females also increased significantly, further confirming the synergistic effect of the three genera. It is presumed that they may participate in intestinal nutrient metabolism and immune regulation in females, and the specific mechanism still needs verification.

### 5.5. Effects of Sex on Metabolic Pathways of Gastrointestinal Microbiota in Small-Tailed Han Sheep

The tissue specificity and sex differences in microbiota structure are ultimately reflected through the functional differentiation of metabolic pathways. the present study found that the level-1 metabolic pathways in both the rumen and colon were dominated by metabolism, genetic information processing, and cellular processes. However, the unique level-1 pathway in the colon–environmental information processing directly reflects the essential difference in their functional positioning: as a specialized “fermentation factory” for ruminants, the rumen focuses on the degradation of carbohydrates (starch, fiber) and fatty acid synthesis to provide the main energy for the host. In contrast, the colon is more involved in amino acid metabolism, degradation of residual polysaccharides/oligosaccharides, and also undertakes the responsibilities of maintaining intestinal homeostasis and immune regulation [62]. Such pathway differentiation is essentially the functional specialization of microbiota driven by the segment-specific physicochemical properties (e.g., pH) and physiological functions of the gastrointestinal tract. Further analysis of level-3 metabolic pathways revealed that carbohydrate metabolism, lipid metabolism, amino acid metabolism, metabolism of other amino acids, metabolism of cofactors and vitamins, metabolism of terpenoids and polyketides, energy metabolism, glycan biosynthesis and metabolism, xenobiotic biodegradation and metabolism, membrane transport, and folding, sorting, and degradation were shared by the rumen and colon, reflecting the core conservation of gastrointestinal metabolism in ruminants. The unique pathways further strengthened the functional differentiation: the unique level-3 pathways in the rumen were concentrated in biosynthesis of other secondary metabolites, transcription, and transport and catabolism, matching its demand for efficient fermentation and energy conversion; The unique pathways in the colon focused on nucleotide metabolism, signal transduction, cell motility, translation, replication and repair, and environmental adaptation, which were highly consistent with the dual functions of the colon as “secondary fermentation of residual substrates + intestinal barrier protection”. This was consistent with the findings of Hu et al. [62] on the functional differentiation between the rumen and colon in ruminants.

In addition to tissue specificity, the regulation of metabolic pathways by sex showed significant targeting, mainly focusing on key pathways closely related to reproductive reserve and growth performance. In the galactose metabolism pathway, the activity in the colon of 6-month-old females was significantly higher than that of males. As an important monosaccharide, galactose can be converted to glucose-1-phosphate via the Leloir pathway to participate in energy metabolism, and is also a direct precursor for lactose synthesis during lactation [71]. This difference is presumed to be related to the physiological development characteristics of females: the ovaries of 6-month-old females have gradually developed, and estrogen levels are higher than those of males. Estrogen can up-regulate the gene expression of key enzymes such as galactokinase (GALK) and UDP-galactose pyrophosphorylase (UGP2) [71]. Even before entering lactation, the galactose metabolism pathway has been strengthened to reserve metabolic capacity for lactose synthesis in milk during the subsequent lactation period, reflecting a reproductive-related metabolic adaptive strategy in females. Another key differential pathway was the one-carbon pool by folate, whose activity was significantly higher in males than in females. As a core intracellular metabolic pathway, it provides a stable methyl donor for DNA synthesis and protein methylation through the folate cycle and methionine cycle, meeting basic metabolic demands such as muscle growth and tissue repair [72]. Meanwhile, it can also improve the body’s antioxidant stress capacity by enriching the one-carbon pool, reducing free radical damage to the liver and muscle tissue [73]. This result is highly consistent with the stronger growth potential and muscle deposition ability of males, as well as their better serum antioxidant phenotype. Essentially, it is metabolic differentiation shaped by sex-specific physiological demands: males focus on supporting basic metabolic efficiency and growth performance by strengthening the one-carbon pool function; females’ one-carbon metabolism tends to adapt to the metabolic flexibility required for subsequent pregnancy and lactation. In conclusion, the core of metabolic pathway differentiation between the rumen and colon of Small-tailed Han sheep is “functional adaptation”, i.e., the rumen focuses on efficient energy supply, while the colon emphasizes homeostasis maintenance and residual substrate utilization. The regulation of metabolic pathways by sex is “physiologically targeted”. Through differential regulation of pathways such as galactose metabolism and folate-mediated one-carbon pool, it adapts to the specific demands of reproductive reserve in females and growth performance in males, respectively, providing a functional basis for optimizing sex-specific feeding schemes for ruminants.

## 6. Conclusions

In conclusion, significant sex differences were detected in slaughter performance and serum biochemical indices of 6-month-old Small-tailed Han sheep. Microbial alpha diversity in the rumen was extremely significantly higher than that in the colon; their community structures were distinctly separated, and the colon presented higher microbial heterogeneity. Sex exerted no significant effect on alpha diversity but showed a tendency toward differences in colonic beta diversity. The influence of sex exhibited tissue specificity: the relative abundances of microbiota at the phylum and genus levels differed significantly between the rumen and colon. Male sheep had a higher abundance of Actinomycota in the rumen, and sex-related differences in colonic microbiota at the genus level were more evident. Bioinformatic prediction of microbial metabolic pathways indicated that the rumen and colon each harbored unique pathways with significant differences in abundance. Rumen and colonic microbiota, as well as their metabolic pathways, underwent significant tissue-specific differentiation. Sex was mainly correlated with production performance, serum biochemical parameters, and specific microbial taxa. The present findings only reveal the correlation between microbiota and host phenotypes, providing a fundamental theoretical basis for sex-specific feeding and management of Small-tailed Han sheep. Given the relatively limited sample size in this study, the conclusions obtained are only phase-based exploratory results, which can serve as a preliminary theoretical reference for the formulation of sex-specific feeding and management strategies in Small-tailed Han sheep. The underlying patterns of related physiological and microecological differences, together with their regulatory mechanisms, still need to be further verified and improved by expanding the experimental population size and conducting multi-site replicated trials in future research.

## Figures and Tables

**Table 1:** Composition and nutritional levels of the diet (dry matter basis).

Traits	Content (%)	Nutrient Levels ^2^	Content (%)
Corn stover silage	50	ME (MJ/kg)	12.84
Corn	25	Crude protein	14.06
Soybean meal	11.5	Ether extract	4.04
Wheat bran	5	Neutral detergent fiber	36.38
Peanut meal	5	Acid detergent fiber	21.19
NaHCO_3_	0.85	Calcium	0.85
Nacl	0.15	Phosphorus	0.61
CaHPO_4_	0.5		
Premix ^1^	2		
Total	100		

^1^ Each kilogram of premix provided the following to the diet: VA 220 kIU, VD 80 kIU, VE 2000 IU, Cu 500 mg, Zn 2500 mg, Mn 2500 mg, I 30 mg, Se 10 mg, Co 35 mg. ^2^ ME was a calculated value, and the others were measured values.

**Table 2:** Slaughter performance of 6-month-old Small-tailed Han sheep of different sexes.

Traits	Female	Male	SEM	*p*-Value
Pre-slaughter live weight (kg)	30.82 ^b^	38.84 ^a^	1.35	0.001
Carcass weight (kg)	15.54 ^b^	19.62 ^a^	0.70	0.001
Net meat weight (kg)	13.18 ^b^	17.14 ^a^	0.66	0.001
Dressing percentage (%)	51.77	51.91	0.34	0.846
Net meat percentage (%)	42.71	44.09	0.37	0.058
Loin eye area (cm^2^)	11.29 ^b^	14.67 ^a^	0.73	0.017
GR value (mm)	12.96	15.74	0.76	0.064
Backfat thickness (mm)	2.68	3.54	0.33	0.200
Tail weight (g)	420.50	555.80	42.71	0.115
Lightness value at 1 h (L*)	29.81	28.83	0.35	0.170
Redness value at 1 h (a*)	17.15	15.98	0.41	0.154
Yellowness value at 1 h (b*)	3.93 ^a^	3.28 ^b^	0.16	0.039
Lightness value at 24 h (L*)	34.23	33.18	0.42	0.224
Redness value at 24 h (a*)	17.93	18.28	0.30	0.568
Yellowness value at 24 h (b*)	6.69	6.13	0.19	0.138
pH value at 45 min	6.13	6.27	0.06	0.267
pH value at 24 h	5.54	5.69	0.05	0.168
Shear force (N)	67.66	73.71	3.88	0.450
Cooked meat percentage (%)	55.58	52.21	1.43	0.249
Drip loss (%)	1.50	1.48	0.05	0.811

In the same row, values with different small letters are significantly different (*p* < 0.05). The animals were divided equally by sex, with 10 females and 10 males.

**Table 3:** Changes in blood biochemical indices of 6-month-old Small-tailed Han sheep of different sexes.

Traits	Female	Male	SEM	*p*-Value
TP (g/L)	67.26	69.74	0.84	0.147
ALB (g/L)	39.08	39.86	0.48	0.430
ALT (U/L)	30.41	31.24	0.47	0.390
AST (U/L)	50.53	50.95	1.18	0.864
TC (mmol/L)	1.70	1.67	0.03	0.702
TG (mmol/L)	0.45	0.43	0.02	0.659
HDL (mmol/L)	1.06	1.04	0.02	0.732
LDL (mmol/L)	0.55	0.54	0.01	0.887
ALP (U/L)	226.62 ^b^	242.98 ^a^	3.52	0.015
BUN (mmol/L)	12.20 ^a^	8.50 ^b^	0.64	0.001
GLU (mmol/L)	8.08	6.58	0.47	0.110
β-BHBA (mmol/L)	0.40 ^a^	0.35 ^b^	0.01	0.003
SOD (U/mL)	104.67	107.31	1.51	0.395
MDA (nmol/mL)	4.45	4.30	0.12	0.573
T-AOC (U/mL)	9.21	9.33	0.18	0.751
GSH-PX (U/mL)	903.45	924.64	10.21	0.314
CAT (U/mL)	11.93	12.41	0.19	0.207

In the same row, values with different small letters are significantly different (*p* < 0.05). The animals were divided equally by sex, with 10 females and 10 males. TP: total protein; ALB: albumin; ALT: alanine aminotransferase; AST: aspartate aminotransferase; TC: total cholesterol; TG: triglyceride; ALP: alkaline phosphatase; BUN: blood urea nitrogen; GLU: glucose; BHBA: β-hydroxybutyric acid; HDL: high-density lipoprotein; LDL: low-density lipoprotein; T-AOC: total antioxidant capacity; GSH-Px: glutathione peroxidase; CAT: catalase; SOD: superoxide dismutase; and MDA: malondialdehyde.

**Table 4:** Changes in serum hormone indices of 6-month-old Small-tailed Han sheep of different sexes.

Traits	Female	Male	SEM	*p*-Value
FFA (umol/L)	385.54	368.41	8.39	0.322
INS (mIU/L)	16.32	13.33	1.55	0.352
T3 (ng/mL)	1.27	1.11	0.11	0.460
T4 (ng/mL)	30.94	29.64	1.24	0.615
CCK (ng/mL)	1.28	1.20	0.05	0.457
GH (ng/mL)	2.88	3.01	0.07	0.364
E2 (pg/mL)	21.51	15.03	1.86	0.080
T (ng/dL)	15.19 ^b^	24.28 ^a^	1.38	0.001

In the same row, values with different small letters are significantly different (*p* < 0.05). The animals were divided equally by sex, with 10 females and 10 males. FFA: free fatty acid; INS: insulin; T3: triiodothyronine; T4: thyroxine; CCK: chole-cystokinin; GH: growth hormone; E2: estradiol; and T: testosterone.

**Table 5:** α-diversity of ruminal and colonic microbiota in 6-month-old Small-tailed Han sheep.

Traits	Rumen	Colon	SEM	*p*-Value
chao1	2635.27 ^a^	1438.35 ^b^	108.54	0.001
observed_species	1803.37 ^a^	1076.48 ^b^	69.83	0.001
PD_whole_tree	140.84 ^a^	84.34 ^b^	5.09	0.001
shannon	8.28 ^a^	6.40 ^b^	0.19	0.001

In the same row, values with different small letters are significantly different (*p* < 0.05). Animals were grouped by tissue/organ: 20 samples for rumen and 20 samples for colon.

**Table 6:** α-diversity of ruminal and colonic microbiota in 6-month-old Small-tailed Han sheep of different sexes.

Traits	Sex	Rumen	Colon	SEM	*p*-Value
chao1	Female	2541.04 ^a^	1428.90 ^b^	148.57	0.001
	Male	2729.51 ^a^	1447.79 ^b^	161.27	0.001
	SEM	76.78	69.04		
	*p*-value	0.229	0.895		
observed_species	Female	1751.49 ^a^	1051.97 ^b^	100.76	0.001
	Male	1855.25 ^a^	1100.98 ^b^	98.56	0.001
	SEM	49.18	60.81		
	*p*-value	0.304	0.698		
PD_whole_tree	Female	138.45 ^a^	82.83 ^b^	7.37	0.001
	Male	143.22 ^a^	85.85 ^b^	7.19	0.001
	SEM	3.01	3.65		
	*p*-value	0.444	0.690		
shannon	Female	8.20 ^a^	6.23 ^b^	0.29	0.001
	Male	8.37 ^a^	6.58 ^b^	0.25	0.001
	SEM	0.08	0.23		
	*p*-value	0.312	0.476		

In the same row, values with different small letters are significantly different (*p* < 0.05). The animals were divided equally by sex, with 10 females and 10 males.

## Data Availability

Raw data for the figures are available upon reasonable request from the corresponding author.

## Abbreviations

The following abbreviations are used in this manuscript:

ALP	Alkaline phosphatase
BUN	Blood urea nitrogen
β-BHBA	β-hydroxybutyric acid
NMDS	Non-metric multidimensional scaling
TMR	Total mixed ration
TP	Total protein
ALB	Albumin
ALT	Alanine aminotransferase
AST	Aspartate aminotransferase
TC	Total cholesterol
TG	Triglyceride
GLU	Glucose
HDL	High-density lipoprotein
LDL	Low-density lipoprotein
T-AOC	Total antioxidant capacity
GSH-Px	Glutathione peroxidase
CAT	Catalase
SOD	Superoxide dismutase
MDA	Malondialdehyde
FFA	Free fatty acid
INS	Insulin
T3	Triiodothyronine
T4	Thyroxine
CCK	Cholecystokinin
GH	Growth hormone
E2	Estradiol
T	Testosterone
